# Deadly intentions: naïve introduced foxes show rapid attraction to odour cues of an unfamiliar native prey

**DOI:** 10.1038/srep30078

**Published:** 2016-07-15

**Authors:** Jenna P. Bytheway, Catherine J. Price, Peter B. Banks

**Affiliations:** 1 School of Life and Environmental Sciences, The University of Sydney, Sydney, NSW 2006, Australia; 2 School of Biological, Earth and Environmental Sciences, The University of New South Wales, Randwick, NSW 2052, Australia

## Abstract

Introduced predators have caused declines and extinctions of native species worldwide, seemingly able to find and hunt new, unfamiliar prey from the time of their introduction. Yet, just as native species are often naïve to introduced predators, in theory, introduced predators should initially be naïve in their response to novel native prey. Here we examine the response of free-living introduced red foxes (*Vulpes vulpes*) to their first encounter with the odour cues of a novel native prey, the long-nosed bandicoot (*Perameles nasuta*). Despite no experience with bandicoots at the study site, foxes were significantly more interested in bandicoot odour compared to untreated controls and to a co-evolved prey, the black rat (*Rattus rattus*). So what gives introduced predators a novelty advantage over native prey? Such neophilia towards novel potential food sources carries little costs, however naïve native prey often lack analogous neophobic responses towards novel predators, possibly because predator avoidance is so costly. We propose that this nexus between the costs and benefits of responding to novel information is different for alien predators and native prey, giving alien predators a novelty advantage over native prey. This may explain why some introduced predators have rapid and devastating impacts on native fauna.

The decline and extinction of native species has repeatedly coincided with the arrival and spread of introduced predators^1^, which have at least double the impact on prey populations as native predators^2^. Introduced predator impacts are often most severe during the acute phase of the invasion process, compared to the chronic phase where native prey may learn and adapt to novel enemies. Hence the initial response of predators and prey to each others’ cues is arguably the most important phase in the predation sequence^3^. The exaggerated impacts of alien predators are usually attributed to a lack of effective anti-predator strategies by native prey towards these predators (naïve prey hypothesis)^4^, which hinges on the assumption that introduced predators enjoy a novelty advantage that facilitates their invasion success^5^. However, given that naiveté towards novel species can go both ways, introduced predators should also be initially naïve to native prey^5^.

Behavioural flexibility is thought to be a key factor in the success of introduced predators, enabling invaders to rapidly respond to novel situations^6^. Such flexibility can include a predilection to consume unfamiliar foods and a positive responsiveness to novelty^7^. When invading a new environment, there is likely to be strong selection for individuals that demonstrate behavioural flexibility to readily switch between prey types and investigate cues of unfamiliar prey, allowing them to quickly become efficient predators of novel prey types. For example, actively invading house sparrows (*Passer domesticus*) are more willing to approach and consume novel foods than individuals in an established population^7^. However, although there are numerous studies on animals rapidly learning about novel food items in a laboratory environment (e.g.^8^,^9^), there is a general lack of understanding of how wild living invasive predators respond to novel prey cues on their initial encounter.

Olfactory cues are crucial to reciprocal recognition in many mammalian predator-prey interactions, yet the role of a shared evolutionary history in the exploitation of odours is not well understood^10^. Field-based experiments on free-living predator responses to evolutionary novel prey cues are an obvious gap in the literature. The few studies that have attempted to fill this niche are predominantly aquatic studies (e.g.^11^) and can not rule out the possibility that predators may have a pre-existing learned associative relationship with the novel prey due to a past encounter.

Here we test the responses of a wild free-living introduced predator, the red fox (*Vulpes vulpes*), to their first encounter with odour cues of a vulnerable Australian native prey species, the long-nosed bandicoot (*Perameles nasuta*). Foxes have been implicated as a major cause of declines and extinctions of Australian critical weight range (CWR; 35–5500 g) native mammals^1^ and five of the other seven bandicoot species in Australia are extinct or critically endangered due to alien predators^12^. Long-nosed bandicoots are coastal CWR mammals that are naïve to foxes^13^ and vulnerable to fox predation, which is known to suppress their populations^14^. We also examine fox responses on first encounter with a co-evolved prey species, the black rat (*Rattus rattus*). Black rats arrived in Australia ca. 200 years ago with settlers from Europe^15^ where they have coexisted with foxes for thousands of years^16^, and are eaten by foxes elsewhere in Australia (e.g. see^17^). Our remote inland study site is well outside the known ranges of both prey species and their close relatives^18^ and so local foxes would have no experience with these species. We test predictions of two alternate hypotheses (1) that foxes will investigate bandicoot and rat odours despite a lack of familiarity (behavioral flexibility hypothesis) or (2) that foxes will not investigate the evolutionary novel bandicoot odours (naiveté hypothesis).

## Results

**Figure 1:**
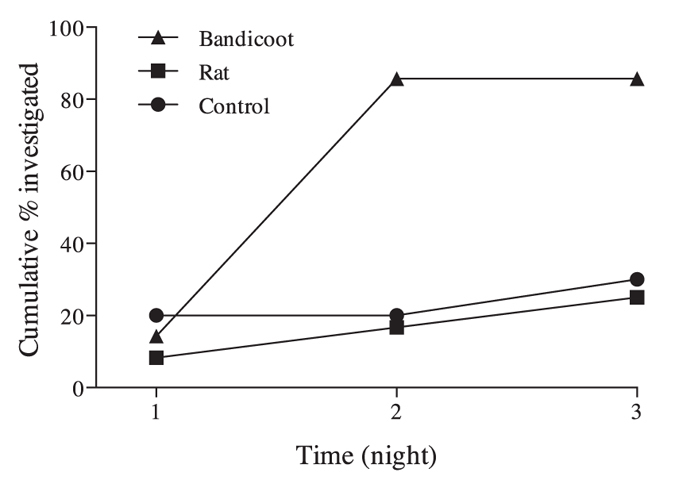
The accumulative percentage of sand plots investigated by foxes over time. Foxes investigated sand plots with bandicoot odours more frequently than sand plots with rat or control odours.

Foxes investigated significantly more sand plots with bandicoot odours than sand plots with rat or control odours (Pearson Chi-Square X^2^ = 7.54, DF = 2 exact sig. (2-sided) *P* = 0.024, adjusted standardized residual of 2.7). More than 80% of sand plots with bandicoot odour were investigated by foxes after two nights, compared to just 17% and 20% of the rat odour and control sand plots respectively (Fig. 1).

## Discussion

Despite no prior experience with bandicoots, foxes showed greater interest in the evolutionary unfamiliar bandicoot odour than to untreated controls, supporting the behavioral flexibility hypothesis. Strong fox interest in the nesting odours of a marsupial species such as bandicoots, which are evolutionary distinct from any prey that foxes have co-evolved with in their native range, may help to explain the success and rapid impacts of introduced foxes in Australia and elsewhere. While foxes used in this study were from established populations and it is possible that they may behave differently on the invasion front, curiosity to novelty is likely to be similar and many of the foxes at our study site would have been dispersing individuals moving through unfamiliar environments. Identifying evolutionary processes of long-established introduced species is difficult. Although there is a remote possibility that the ancestors of the foxes at our study site could have encountered bandicoot sp. as they spread from their release site near Melbourne, Victoria in the 1870s^19^, it is unlikely. Foxes spread rapidly across Victoria after release, arriving in Walpeup within approximately 20 years^19^, limiting the opportunity for genetic selection and retention of specific recognition of bandicoot odour. Furthermore, although there is speculation that other bandicoots species might have once occurred in this region, their exact distributions are unknown^20^. We argue that it seems more likely that the rapid attraction to bandicoot odour by foxes reflects a general predator strategy, particularly of introduced predators, to be attracted to novelty. So what gives introduced predators the novelty advantage over native prey?

**Figure 2:**
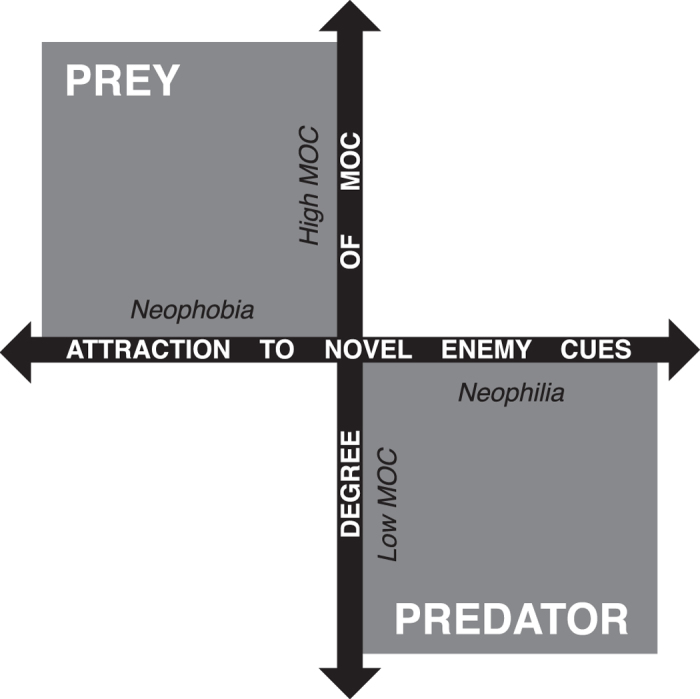
Neophilia-neophobia nexus: response strategies of predators and prey to the initial encounter with cues of the other, and the associated missed opportunity costs (MOC). Predators should be attracted to prey cues and pay low MOC for doing so, whereas if prey respond to predator cues, this response carries high MOC.

Investigating unfamiliar prey cues (neophilia) can carry relatively little cost to introduced predators for a potentially large reward; if investigation is rewarded, predators can rapidly develop search images^21^ that can be devastating for naïve prey. In contrast, native prey may suffer considerable missed opportunity costs should they forgo foraging by performing unnecessary anti-predator responses upon initial encounter with unfamiliar predator cues (neophobia^22^) and will rapidly habituate to cues of risk that are not realised^23^. We propose that this represents a neophilia-neophobia nexus (Fig. 2) reflecting an imbalance in the cost:benefit relationship for alien predators and native prey responding to novel information about their enemy. In time, naïve prey can develop refined recognition and responses to novel predators^4^ however alien predators displaying neophilia skip over this initial learning stage, giving them an advantage, which may help to explain why so many native species rapidly decline after the arrival of alien predators. Our results provide preliminary support for this model, however it should be considered as a theoretical basis for future experimental testing.

That foxes showed greater interest in bandicoot than rat odours was surprising. The lack of fox interest in the odours of black rats may be because they were specifically recognised as an unprofitable prey. Black rats likely possess more effective anti-predator strategies against foxes compared to bandicoots given their shared evolutionary history, making them a more difficult prey option. It is also possible that attraction to the evolutionary novel prey may reflect a general inherent preference for novel prey by alien predators. Novel prey are likely to be naïve to foxes and have ineffective anti-predator responses^5^. There is some support for this idea: foxes continued to depredate an already reduced population of bettongs (another Australian CWR marsupial) amongst a high-density rabbit (*Oryctolagus cunniculus*) population (an evolutionary familiar prey)^24^.

The exaggerated impacts of introduced predators on native prey are commonly attributed to shortcomings in native prey^5^. However the outcome of a predator-prey interaction is determined by tactics of both predators and prey^3^. Our results suggest that neophilic tactics of alien predators towards novel information during initial encounters with potential prey may tip the novelty advantage towards predators. Alien predator impacts will be greatest when these neophilic tactics occur during the acute phase of invasion before native prey can develop effective anti-predator tactics. Innovative management strategies are needed to undermine this introduced predator novelty advantage (e.g.^25^).

## Methods

### Odour collection

Odours indicative of nesting long-nosed bandicoots and black rats were used, as both species rest in den sites and are vulnerable to olfactory predators^14^,^26^. Animal odours (body, urine, faeces) were collected on towels (20 × 30 cm) placed in cage traps for one night at North Head, Sydney, Australia. Scented towels were sealed in airtight bags and stored at −20 °C which is unlikely to affect odour degradation^23^. Controls were clean towels, and separate gloves were worn when handling samples to prevent cross-contamination of odours.

### Experimental method

Seventy-two sand monitoring plots (2 m wide) were constructed >500 m apart using *in situ* sand across dirt roads over a 225 km^2^ area of agricultural land and remnant mallee woodland at Walpeup, north-western Victoria, Australia (>800 km from where odours were collected and >300 km from the nearest bandicoot record; rats can occur in major rural centres, for example Mildura, but rarely outside^27^).

Foxes were habituated to the experimental set-up to enable separation of initial responses to the set-up from responses to odour treatments. This involved a familiarisation phase whereby a clean towel (20 × 30 cm) was pinned immediately adjacent to the edge of the sand plot with a tent peg and lightly covered with *in situ* sand to reduce visual conspicuousness. Foxes within the vicinity of a sand plot were considered to have familiarised to the plot if the level of interest in the clean towel decreased after 4–13 days.

**Figure 3:**
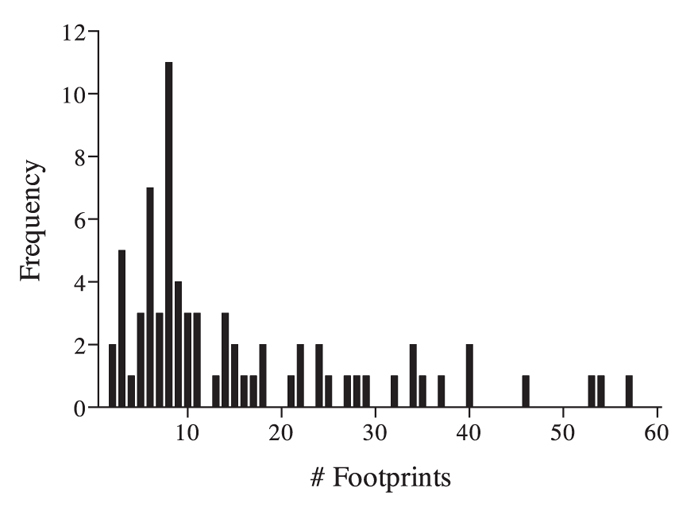
Frequency histogram of the number of fox footprints recorded within one metre of the towel. A distinct peak at eight footprints was indicative of an incidental passing.

**Figure 4:**
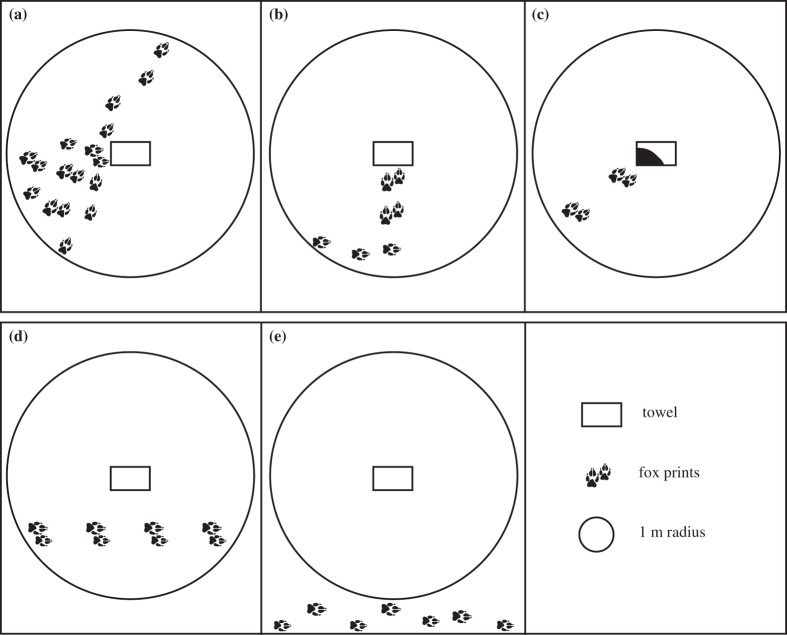
Representation of arrangement of fox footprints considered an investigation and incidental passing. Investigation: (**a**) >10 footprints within 1 m of the towel, (**b**) obvious change in direction, (**c**) diggings at towel. Incidental passing: (**d**) ≤10 footprints, (**e**) no footprints within 1 m of the towel.

Fox activity on the sand plot was classified as an ‘investigation’ of the experimental equipment based on the spatial arrangement, direction and concentration of footprints within one metre of the towel, (derived from a frequency histogram (Fig. 3); a distinct peak at eight footprints was indicative of an incidental passing i.e. the number of tracks taken to cross the sand pad in a straight line). An ‘investigation’ of the equipment met one of the following criteria: more than ten fox footprints or an obvious change in direction of footprints within one metre of the towel, or diggings at the towel (Fig. 4). All other fox activity on the sand plot was classified as an ‘incidental passing’.

It was assumed that interest in the experimental set up had dropped if fox activity declined from being classified as investigations to incidental passing’s or if a plot was repeatedly passed without being investigated. Following these criteria, we considered foxes had become familiar to the experimental equipment at 40 plots.

All plots received treatments on the same day, which involved pegging a treatment towel (long-nosed bandicoot odour, black rat odour, or control) to the edge of the sand plot as in the familiarisation phase. Sand plots were monitored and re-raked daily for three days and an observer blinded to the odour treatments identified all animal prints present. Fox activity was classified as either an investigation or incidental passing as above (Fig. 4). Only plots where foxes had familiarised to the setup and then recorded fox activity during the three treatment nights were included in the analyses (n = 7, 12 and 10 for bandicoot, rat and control treatments respectively).

### Statistical analyses

We used a Chi-Square test in PASW Statistic 18 using exact probability values followed by adjusted standardised residuals to identify treatment effects; values greater than two indicate a lack of fit to the null hypothesis in that cell^28^. Analyses were conducted on the total number of plots that had recorded fox activity after three nights. Plots were scored as investigated if they recorded a fox investigation on any of the three treatment nights, regardless of other visits (incidental passings) to that plot.

### Ethics statement

All field methods were carried out in accordance with procedures that were approved by the University of New South Wales Animal Ethics Committee (08/153B).

## Additional Information

**How to cite this article**: Bytheway, J. P. *et al*. Deadly intentions: naïve introduced foxes show rapid attraction to odour cues of an unfamiliar native prey. *Sci. Rep.* **6**, 30078; doi: 10.1038/srep30078 (2016).
